# Editorial for the Special Issue “Hydrogels for 3D Printing”

**DOI:** 10.3390/gels10050323

**Published:** 2024-05-09

**Authors:** Enrique Aguilar, Helena Herrada-Manchón

**Affiliations:** 1 Centro de Innovación en Química Avanzada (ORFEO-CINQA), Departamento de Química Orgánica e Inorgánica, Instituto Universitario de Química Organometálica “Enrique Moles”, Universidad de Oviedo, C/Julián Clavería 8, 33006 Oviedo, Spain; 2 Fundación Idonial, Parque Científico y Tecnológico de Gijón, Avda, Jardín Botánico 1345, 33203 Gijón, Spain

Hydrogels, which are three-dimensional networks of hydrophilic polymers capable of absorbing and retaining large amounts of water, have emerged as versatile materials with vast potential in various fields. In the context of 3D printing, hydrogels offer unique advantages due to their tunable mechanical properties, biocompatibility, and ability to encapsulate cells or bioactive molecules. The process of 3D printing with hydrogels comprises several crucial steps, each of which plays a critical role in determining the final structure and properties of the printed object.

A key aspect in this process is the formulation of hydrogel materials used for printing. The selection and optimization of raw materials or components are crucial to ensure the desired rheological properties and printability of the biomaterial ink or bioink. During printing, the hydrogel material is deposited layer by layer, following the path determined by slicing software. The rheological properties of the hydrogel formulation are essential at this stage to ensure the proper deposition of the material and maintain the structural integrity of the printed object.

In addition to rheological considerations, the choice of hydrogel formulation must consider the specific requirements of the intended application. For instance, in tissue engineering applications, hydrogels must provide a supportive matrix for cell growth and tissue regeneration. Therefore, factors such as biocompatibility, cell adhesion, and degradation kinetics are of utmost importance.

Furthermore, post-processing steps such as crosslinking are often necessary to stabilize the printed structure and enhance its mechanical properties. Crosslinking agents, such as chemical or physical crosslinkers, are used to form covalent or physical bonds between polymer chains, resulting in a more robust and stable hydrogel network.

This Special Issue showcases a diverse array of seven articles and three reviews that explore the multifaceted aspects of hydrogels for 3D printing, encompassing all the aforementioned steps, as follows:

1. The review “Essential Guide to Hydrogel Rheology in Extrusion 3D Printing: How to Measure It and Why It Matters?” by Herrada-Manchón et al. examines the crucial role of rheology in extrusion-based 3D printing, focusing on hydrogels and their applications in tissue engineering, regenerative medicine, and drug delivery. Understanding rheological properties such as shear-thinning behavior, thixotropy, and viscoelasticity is essential for optimizing the printing process and achieving the desired product quality.
2. The review “3D-Printed Hydrogel for Diverse Applications: A Review” by Agrawal et al. explores the intersection of hydrogels and 3D printing, covering current research, technological advancements, and future directions in various applications, including biomedical engineering. It discusses hydrogel basics, materials, 3D printing methods, and challenges, in addition to predicting future trends.
3. The review “Biopolymers for Tissue Engineering: Crosslinking, Printing Techniques, and Applications” by Patrocinio et al. explores the use of proteins and polysaccharides in bioprinting for tissue engineering, focusing on their biocompatibility, biodegradability, and biomimicry. It addresses challenges related to rheological behaviors and the need for modifications or crosslinking. The manuscript also discusses tissue engineering applications, crosslinking, and bioprinting techniques, aiming to achieve bioprinted structures mimicking extracellular matrix properties with good levels of printability and stability.
4. The article “Microbial Polysaccharide-Based Formulation with Silica Nanoparticles; A New Hydrogel Nanocomposite for 3D Printing” by Marin et al. develops printable hydrogel nanocomposites by adding silica nanoparticles to a microbial polysaccharide polymer network, aiming for biomedical applications. Their morpho-structural characteristics, swelling behavior, and mechanical stability were assessed, revealing excellent biocompatibility, making them suitable for regenerative medicine.
5. The article “Three-Dimensional Printing of Red Algae Biopolymers: Effect of Locust Bean Gum on Rheology and Processability” by Oliveira et al. explores the extraction of gelling biopolymers from red seaweeds to produce sustainable food gels and assess their potential as bioinks for 3D printing, focusing on improving gel matrix definition through the addition of locust bean gum, as well as adjusting printing temperature.
6. The article “A New Method for the Production of High-Concentration Collagen Bioinks with Semiautonomic Preparation” by Matejkova et al. introduces a novel method for preparing highly concentrated collagen bioinks, utilizing a two-step neutralization process based on bicarbonate buffering mechanisms and pH adjustment. The automated bioink preparation process ensures consistent quality, demonstrating sustained cell proliferation and viability, in addition to offering potential for advancing tissue engineering applications.
7. The article “Alginate–Gelatin Hydrogel Scaffolds; An Optimization of Post-Printing Treatment for Enhanced Degradation and Swelling Behavior” by Kaliampakou et al. addresses the optimization of the post-printing treatment phase in 3D structure generation, focusing on enhancing scaffold degradation while maintaining targeted swelling behavior.
8. The article “Characterization of Bioinks Prepared via Gelifying Extracellular Matrix from Decellularized Porcine Myocardia” by Sanz-Fraile et al. introduces a novel cardiac bioink derived exclusively from decellularized porcine myocardium and loaded with human-bone-marrow-derived mesenchymal stromal cells, eliminating the need for additional biomaterials or crosslinkers.
9. The article “Role of pH and Crosslinking Ions on Cell Viability and Metabolic Activity in Alginate–Gelatin 3D Prints” by Souza et al. investigates the influence of pH and crosslinking ions on the stability, printability, and cell behavior of alginate–gelatin hydrogels commonly used in bioengineering. The results reveal that buffer pH and crosslinking ions affect the swelling and degradation rates of prints, as well as hydrogel printability.
10. The article “Polydopamine Blending Increases Human Cell Proliferation in Gelatin–Xanthan Gum 3D-Printed Hydrogel” by Yerra et al. enhances the printability of gelatin–xanthan gum hydrogel by incorporating polydopamine (PDA), a mussel-inspired biopolymer, to create cell-laden 3D scaffolds demonstrating improved fibroblast and keratinocyte growth without affecting hydrogel characteristics, thereby suggesting its potential for developing innovative 3D-printed wound dressings.

The continued exploration of hydrogels for 3D printing holds promise for groundbreaking advancements in areas such as tissue engineering, drug delivery, and regenerative medicine. We extend our gratitude to the authors for their valuable contributions to this Special Issue and look forward to the transformative impact of their work.

